# High Prevalence of* Leptotrichia amnionii*,* Atopobium vaginae*,* Sneathia sanguinegens*, and Factor 1 Microbes and Association of Spontaneous Abortion among Korean Women

**DOI:** 10.1155/2017/5435089

**Published:** 2017-12-13

**Authors:** Sang Soo Seo, Selvaraj Arokiyaraj, Mi Kyung Kim, Hea Young Oh, Minji Kwon, Ji Sook Kong, Moon Kyung Shin, Ye Lee Yu, Jae Kwan Lee

**Affiliations:** ^1^Division of Cancer Epidemiology and Management, Center for Uterine Cancer, National Cancer Center, Ilsandong-gu, Goyang, Republic of Korea; ^2^Asan Medical Center, University of Ulsan College of Medicine, Seoul, Republic of Korea; ^3^Department of Obstetrics and Gynecology, Korea University College of Medicine, Seoul, Republic of Korea

## Abstract

*Objective. *The purpose of this study was to (i) determine the cervical microbial composition in different abortion samples and to (ii) investigate the correlation between spontaneous abortion and cervical microbes in Korean women.* Methods. *We collected cervical swabs from women who had never undergone abortion (*N* = 36), had spontaneous abortion (*N* = 23), and had undergone induced abortion (*N* = 88) and subjected those samples to 16S rRNA pyrosequencing. Further, factor analysis and correlation between cervical microbiota and spontaneous abortion were evaluated by logistic regression analysis.* Results. *In spontaneous abortion women, 16 S rRNA gene sequences showed significant increases in* Atopobium vaginae*,* Megasphaera* spp.,* Gardnerella vaginalis*,* Leptotrichia amnionii*, and* Sneathia sanguinegens* compared to women in nonabortion group. In multivariate logistic regression analysis,* A. vaginae* (OD = 11.27; 95% = 1.57–81)*, L. amnionii* (OD = 11.47; 95% = 1.22–107.94),* S. sanguinegens* (OD = 6.89; 95% = 1.07–44.33), and factor 1 microbes (OD = 16.4; 95% = 1.88–42.5) were strongly associated with spontaneous abortion.* Conclusions. *This study showed a high prevalence of* L. amnionii, A. vaginae, S. sanguinegens*, and factor 1 microbes in spontaneous abortion and association with spontaneous abortion in Korean women.

## 1. Introduction

Cervical microbiota play a prominent role in women's reproductive health, which is influenced by numerous factors including age, ethnicity, genetic factors, cultural and economic factors, personal hygiene, sexual activity, and environmental conditions [1, 2]. Cervical microbiota are liable to change throughout a woman's lifetime (birth, puberty, and menopause) [3]. Such changes in vaginal microbial flora have serious consequences such as prevention of fertilization and induction of spontaneous abortion in pregnant women, as well as increased risk of preterm birth and low birth weight [4–8]. Spontaneous abortion prior to 20 weeks is a common adverse outcome of pregnancy. Moreover, spontaneous abortion and other adverse pregnancy outcomes have been traced to be associated with bacteria and viruses infection [9, 10]. A recent study using next-generation sequencing (NGS) techniques demonstrated a vaginal microbiota difference between preterm delivery and normal spontaneous delivery [11], and also there is evidence on cervical microbiota associations with pelvic inflammatory disease, infertility, cervical intraepithelial neoplasia, and obesity [12–15]. However, the epidemiological data on the potential association between cervical microbiota and spontaneous abortion has been rarely reported and is lacking. The objective of this study was to (i) determine the cervical microbial composition in different abortion samples and to (ii) investigate the correlation between spontaneous abortion and cervical microbes in Korean women.

## 2. Methods

### 2.1. Subject Selection and Sample Collection

This study was approved by the Institutional Review Board of the National Cancer Center (IRB numbers NCCNCS-06-062 and NCCNCS 2016-0147). Written informed consent was obtained from all participants. We confirm that all experiments were performed in accordance with relevant guidelines and regulations. The study included women between 18 and 65 years of age who had participated in the Korean Prospective Study of the Transition of Human Papillomavirus into Cervical Carcinoma from 2006 to 2013 [16, 17]. These women were randomly selected from the Gynecology and Oncology clinic in six University hospitals, in South Korea. Eligible women were currently sexually active or seeking birth control, not currently pregnant, and had an intact uterus and no personal history of cervical intraepithelial neoplasia within 18 months. The exclusion criteria were women with a history of cervical cancer, incomplete questionnaire, inadequate blood sample, chronic diseases (liver cirrhosis, renal failure), cardiovascular disease, drug dependency, or psychological problems. Regarding the history of abortion, subjects were classified into 3 groups: 36 nonabortion, 23 spontaneous abortions, and 88 induced abortions among a total of 147 subjects with cervical swab samples.

### 2.2. Questionnaires Related to History of Abortion

Detailed interviewer-administered comprehensive health and lifestyle questionnaires, including questions on behavior related to abortion, such as history of spontaneous abortion and induced abortion and the number of each type of abortion, were completed at enrolment in the outpatient Department of Gynecology and Oncology clinic. The questionnaire included reproductive (menarche age, the number of pregnancies, the number of childbirths, gestational age, and breast feeding) and menstrual history (menopausal status); exogenous hormone use before pregnancy and after menopause, medical history, family history of cervical cancer, and sociodemographic and lifestyle characteristics were recorded. Pathological and laboratory data were collected, recorded, and entered into the epidemiological database, National Cancer Center. Medical charts and pathology reports were examined to insure that control subjects had no history of any cancer or precancerous lesions.

### 2.3. HR-HPV DNA Detection and Pap Smear

Upon study entry, the participants underwent a physical and gynecological examination and had Hybrid Capture 2 testing and Papanicolaou (Pap) smears. The cervical cytological findings were classified according to the Bethesda system [18]. Cervical samples were collected using a Cervix brush (Rovers Medical Devices, Oss, the Netherlands), and the brush was immediately rinsed in a vial of PreservCyt solution (Cytyc Corporation, Marlborough, MA, USA), and the vial was placed in a Thin Prep (Cytyc Corporation, Marlborough, MA, USA) Processor. Collected samples were stored at −80°C for further analysis. The chemiluminescent HPV DNA test yielded relative light units (RLU) using a probe designed to detect 13 HR-HPV types. HPV DNA detection was performed with the Digene HC2 high-risk DNA test (Qiagen, Gaithersburg, MD, USA) with signal amplification and chemiluminescence for detection of 13 types of HR-HPV scored in relative light units (RLU). The test results were read as positive at concentrations of 1 pg/ml or levels greater than the RLU/cutoff ratio (RLU of the specimen/mean RLU of 2 positive controls).

### 2.4. DNA Extraction and Pyrosequencing

Genomic DNA was extracted from the cervical samples by Fast DNA SPIN extraction kits (MP Biomedicals, Santa Ana, CA, USA) following manufacturer's instruction. Isolated DNA from cervical samples was used as template to amplify V1–V3 regions using bar-coded primers. The PCR reaction was performed in a final volume of 50 *μ*L containing 10x Taq buffer, a dNTP mixture (Takara, Japan), 10 *μ*M of the bar-coded fusion primers, and 2 U of Taq polymerase (ExTaq, Takara). The PCR program was as follows: initial denaturation (94°C for 5 min), product amplification, 30 cycles (30 s, 94°C), primer annealing (30 s, 55°C), and extension (30 s, 72°C), followed by a final extension for 7 min at 72°C. The amplified product was checked by 2% agarose gel electrophoresis and visualized under a Gel Doc system (Bio-Rad). The amplified products were purified with a QIAquick PCR purification kit (Qiagen, Valencia, CA, USA) and quantified using a PicoGreen dsDNA Assay kit (Invitrogen, Carlsbad, CA, USA). Equimolar concentrations of each amplicon from different samples were pooled and purified using an AMPure bead kit (Agencourt Bioscience, Beverly, MA, USA) and then amplified on sequencing beads by emulsion PCR. The beads recovered following emulsion PCR were deposited on a 454 Pico Titer Plate, and sequencing was performed using a Roche/454 GS Junior system (Roche, Branford, CT, USA). Raw MiSeq reads were demultiplexed according to the barcodes and trimmed by in-house Perl scripts for quality filtering (quality score > 25). The processed paired reads were assembled, and the assembled reads were used for operational taxonomic unit picking. Quantitative Insights into Microbial Ecology was used for the microbial community analysis. In order to confirm the richness and diversity of the bacterial types in the samples, the Chao1 and Shannon indices were calculated. The 16S rRNA gene sequences obtained from pyrosequencing have been available at the EMBL SRA database (http://www.ebi.ac.uk/ena/data/view/PRJEB5760).

### 2.5. Statistical Analysis

The distributional differences of the continuous and categorical variables among the groups were examined by *t*-test or ANOVA and chi-square test, respectively. To compare the relative abundance differences of microbes among the three abortion groups, we used Wilcoxon rank sum test. Factor analysis was performed to identify the microbial patterns of 45 microbial species (filtered by a 0.1%-or-over rate in individual proportions) using the FACTOR PROCEDURE in SAS (version 9.4; SAS Institute, Chicago, IL, USA). The factors were rotated by an orthogonal transformation (Varimax rotation function in SAS) to achieve a simpler structure with greater interpretability. After the Varimax rotation, the factor scores were saved from the principal component analysis for each individual. All of the data presented here are from the Varimax rotation. Rank correlation analysis between the microbiota and epidemiological factors was performed by Somers' *D* multiple comparison test. Multivariate logistic regression analysis was performed after adjustment for age, BMI, menopausal status, alcohol drinking, smoking habit, and HR-HPV infection. The strength of the association of selected microbes and different abortion groups were reported as the odds ratio (OR) and 95% CI compared to a reference group.

## 3. Results

Table 1 lists the study participants' general characteristics, which include the following epidemiological factors: age, body-mass index (BMI), marital status, menopausal status, number of children, education level, family income, oral contraceptive use, smoking status, alcohol-drinking status, and oncogenic high-risk human papillomavirus (HR-HPV) infection. The participants' mean age was 44 years, and 65% were premenopausal. Significant differences in age, education level, and HR-HPV were observed in the spontaneous abortion group relative to the nonabortion group.

After quality control, a total of 1 431 278 valid reads were obtained, and an average of 92.2 operational taxonomic units (OTUs) per sample were observed by 16S rRNA pyrosequencing analysis. The sequence reads were assigned to 101 OTUs in the nonabortion group, 93 OTUs in the spontaneous abortion group, and 90 OTUs in the induced abortion group. The diversity (Shannon) and richness estimation (Chao 1) were used for measuring alpha diversity. The observed mean values of the Shannon indices and Chao 1 were 1.9 and 118.2 for nonabortion, 2.0 and 111.2 for spontaneous abortion, and 1.9 and 113.2 for induced abortion (Supplementary Figure  1 in Supplementary Material, available online at https://doi.org/10.1155/2017/5435089). Among abortion samples, the spontaneous abortion women showed high diversity and low richness compared to nonabortion women. As for the bacterial communities, taxonomic classification revealed that Firmicutes (73.5%, 54.0%, and 66.5%) was the most dominant phylum followed by Actinobacteria (8.72, 29.21, and 10.31%), Bacteroidetes (4.21, 9.03, and 8.51%), Proteobacteria (8.06, 3.28, and 5.30%),* Fusobacteria* (0.11, 2.48, and 3.49%), and Tenericutes (2.73, 1.02, and 4.00%) in nonabortion, spontaneous abortion, and induced abortion, respectively. The abundances of Firmicutes, Proteobacteria, and Tenericutes were lower in spontaneous abortion than in nonabortion.

Next, we investigated whether the relative abundances (>0.1%) of the cervical microbiota differed between abortion and nonabortion at the species level by 16S rRNA sequencing. We observed that women who had spontaneous abortion showed significant increases (*P* < 0.05) in* Atopobium vaginae*,* Megasphaera* spp.,* Gardnerella vaginalis*,* Leptotrichia amnionii*, and* Sneathia sanguinegens* compared to nonabortion group (Table 2). Also,* Lactobacillus crispatus* were higher in nonabortion, but not statistically significant (Supplementary Table  1). Further, the microbes which showed significant results are selected for logistic regression analysis (univariate and multivariate).

In factor analysis, a total of seven factors showing an eigenvalue greater than 1.5 were identified, and, for each, the factor-loading values of 45 species were provided (Table 3). Among the factors, factor 1 scored a high eigenvalue and was selected for logistic regression analysis. The high-scoring factor 1 microbes included* Megasphaera* sp. (0.711),* P. amnii *(0.528),* P. timonensis* (0.414),* L. amnionii* (0.400),* A. vaginae* (0.259),* S. sanguinegens *(0.208), and* D. microaerophilic* (0.146); the lowest-scoring factor 1 microbes were* L. gasseri* (−0.039),* L. acidophilus* (−0.025),* L. crispatus *(−0.298),* L. vaginalis* (−0.191),* L. fornicalis* (−0.133),* L. jensenii* (−0.115), and* L. psittaci* (−0.13). High eigenvalues also were observed for factors 4, 5, and 7, but these were not included in the subsequent logistic regression analysis; rather, they were used for comparison with the epidemiological factors in estimating the associations with abortion.

Next, rank correlation between epidemiological factors and the relative abundances of 45 microbial species (>0.1%) were conducted (Table 4). We noticed high positive associations between spontaneous abortion and* A. vaginae* (*r* = 0.335; *P* = 0.026),* S. sanguinegens* (*r* = 0.311; *P* = 0.009),* G. vaginalis* (*r* = 0.286; *P* = 0.045), and* L. amnionii* (*r* = 0.265; *P* = 0.024). On the other hand, negative correlations were shown with education, first parity age, and* L. acidophilus*. However, income and education level were positively associated with* P. bivia* and* Lactobacillus* sp. The other epidemiological factors showed only rare correlations with microbial species.

We assessed association between the selected microbes and spontaneous abortion using logistic regression model (Table 5). Based on the univariate analysis there was strong association between spontaneous abortion and high tertile* A. vaginae*, 5.38 (95% CI 1.68–17.29),* S. sanguinegens*, 5.14 (95% CI 1.35–19.54),* L. amnionii*, 4.26 (95% CI 1.11–16.42), and factor 1 microbes, 4.51 (95% CI 1.41–14.45). As for the multivariate analysis, the ORs were calculated after adjustment for age, menopause, BMI, smoking, alcohol, and HPV as categorical variables:* L. amnionii* 11.47 (95% CI 1.22–107.94),* A. vaginae* 11.27 (95% 1.57–81.0), and* S. sanguinegens* 6.89 (95% CI 1.07–44.33) were found to be associated with spontaneous abortion, and factor 1 microbes also showed a high score 16.4 (95% CI 1.88–42.5). The associations between* Megasphaera* spp. and* G. vaginalis* and spontaneous abortion were found to be nonsignificant. In the present study, the number of women using hormone replacement therapy (HRT) was small: 6 in nonabortion, 4 in spontaneous abortion, and 14 in induced abortion group. After excluding women with using HRT, the logistic result was almost the same (data not shown).

## 4. Discussion

By comparing the cervical microbiota profiles of different abortion groups, we found higher prevalence of cervical microbes such as* L. amnionii*,* A. vaginae*,* S. sanguinegens*, and factor 1 microbes in spontaneous abortion women and showed strong association with spontaneous abortion. In the general characteristics of our study subjects among the three abortion groups, we found significant differences in age, education, and oncogenic HPV infection rate between women who had spontaneous abortion and those who had undergone induced abortion (Table 1). With respect to age, we found, similarly to the result of Gracia et al. [19], that the highest percentage of spontaneous abortion cases (43.5%) was that of individuals within the 45–54 age range. Also, among those whose education ended at high school, the prevalences of spontaneous abortion and induced abortion were relatively high compared with that of the nonabortion group (Table 1). Our results agree with previous reports, which mention that spontaneous abortion women were more likely to be unmarried and to have had less education and a history of abortion and pelvic inflammatory disease [20]. Bacteria such as* Lactobacillus fornicalis*,* L. jensenii*, and* L. vaginalis* showed positive correlations with education groups (Table 4). A study conducted by Conde-Ferraez et al. reported that HPV infection was not significantly associated with spontaneous abortion [21]. But, in our study, we found that oncogenic HR-HPV infection was significantly associated with spontaneous abortion (*P* < 0.05). The possible mechanisms of spontaneous abortion by HPV infection are not yet clear, but there is already some evidence that HPV induces apoptosis of infected trophoblasts, thereby negatively affecting implantation and placental physiology [22–25].

At the phylum level, the predominant cervical microbiota of each group was Firmicutes*, Actinobacteria, Bacteroides, Proteobacteria*,* Tenericutes*, and* Fusobacteria*. These observed relative abundances are similar to those observed in our earlier analysis of cervical microbiota in CIN samples [15]. Also, the OTUs were higher in the nonabortion group than in the spontaneous and induced abortion samples. We hypothesize that women in the spontaneous abortion group, relative to those in the nonabortion group, might have had cervical microbial dysbiosis, or that there were other factors such as the sample collection method, sequencing method, hygiene, glycogen level, menstrual cycle, or host-genetic factors [26–28]. It is well known that healthy women harboring high numbers of* Lactobacillus *sp. produce lactic acid and hydrogen peroxide, thus preventing or suppressing the entry of* G. vaginalis, Mobiluncus sp., Prevotella*, and* Bacteroides* that are associated with BV [29, 30]. In this study, we found low relative abundances of* L. inners, L. crispatus*, and* L. johnsonii* and higher abundances of* A. vaginae, A. christensenii, L. amnionii, P. amnii, L. fornicalis, U. parvum, M. hominis*, and* S. sanguinegens* in spontaneous abortion women when compared with women without abortion history. These pathogens are reported to be associated with adverse pregnancy outcomes such as preterm delivery, abortion, chorioamnionitis, and BV [31–33]. Similarly, Dasari et al. reported that low* Lactobacillus* population is associated with reduced vaginal-secretion leukocyte protease inhibitor and increased abnormal flora [34]. Additionally, the relative abundances of unclassified bacteria (AY958888, AY959109, and AY958940) significantly increased in spontaneous abortion compared with nonabortion women (Supplementary Table  1). To better characterize these unknown bacteria and their role in abortion, further studies are required.

Several studies have already established the correlation of high-load* A. vaginae* and* G. vaginalis* with preterm birth [35]. In this study, we found that* L. amnionii, A. vaginae*,* S. sanguinegens*, and factor 1 microbes are highly associated with spontaneous abortion (Table 5). The bacteria* L. amnionii *can be characterized as anaerobic, gram-negative, and pleomorphic coccobacillus found in the oral cavity and genital tract [36]. A recent Norwegian case study of* L. amnionii* was the first to isolate* L. amnionii* from renal abscess in spontaneous abortion patients with chorioamnionitis [37]. Although* L. amnionii *is associated with spontaneous abortion, the epidemiological evidence of an association of high-risk cervical microbiota with spontaneous abortion is lacking.* S. sanguinegens* is a gram-negative, anaerobic, nonmotile, and non-spore forming bacteria found in the gastrointestinal and female genital tracts. This bacteria is reported to be associated with bacterial vaginosis, a vaginal disorder in women of reproductive age worldwide, and is the most common genera detected in amniotic fluid; its presence can lead to inflammation, histological chorioamnionitis, and/or amnionitis [38, 39]. Some studies have demonstrated the phylogenetic relationship between* L. amnionii *and Sneathia, where the former has been assigned to the genus* Sneathia* [40]. The presence of* A. vaginae* is even associated with spontaneous abortion.* A. vaginae* is a species of gram-positive, rod-shaped anaerobic bacteria that can lead to maternal sepsis and spontaneous abortion [41]. Our hypothesis, therefore, is that high proportions of* L. amnionii*,* S. sanguinegens*,* A. vaginae*, and factor 1 microbes in the vagina can increase the likelihood of uterine, fetal-membrane, or fallopian-tube infection leading to spontaneous abortion. Though other notable organisms like* Candida* species and* Trichomonas vaginalis* are also associated with spontaneous abortion [42], the targeted 16S rRNA in the current experiment could not reveal their significant presence.

Strengths of our study are as follows. (1) To our knowledge, this is the first large-cohort study to explore the relative abundances of species associated with the cervical microbiota of women who had never undergone abortion, had spontaneous abortion, or had undergone induced abortion. (2) The use of the pyrosequencing method for identification of fastidious or uncultivable microbes reduced the bias/error as compared with cultivation-based microbiological methods.

However, we recognize certain limitations in this study. (1) Since the abortion status was determined using questionnaires, and the microbiome analysis of the cervical swabs was performed at enrolment, the timings of the event and swab sampling were not the same. This might have biased the findings of the present study. However, further studies are required to understand the importance of association between cervical microbial community and HPV persistence.

(2) ~35% of the participants in the current experiment were postmenopausal; the correlation of microbiome profiles of pre- and postmenopausal participants with the abortion might vary significantly. (3) The small sample size might have limited the significance of the obtained results; however, it was certainly adequate for this study's preliminary determination: that* L. amnionii, A. vaginae, S. sanguinegens*, and factor 1 microbe prevalence are highly associated with spontaneous abortion.

## 5. Conclusion

This study compared the cervical bacterial communities of different abortion women and revealed the association of high* L. amnionii*,* A. vaginae,* S*. sanguinegens*, and factor 1 microbes with spontaneous abortion.

## Supplementary Material

Supplementary table 1: Distribution of vaginal bacteria from non-abortion, spontaneous abortion and induced abortion women in Korea.
Supplementary figure 1: Shannon index (a) and Chao 1 (b) index across non-abortion, spontaneous abortion and induced abortion women.

## Figures and Tables

**Table 1:** General characteristics of study subjects by abortion status: nonabortion, spontaneous abortion, and induced abortion.

	Total	Nonabortion^3^	Spontaneous abortion	Induced abortion	*P* ^4^	*P* ^5^
	(*N* = 147)	(*N* = 36)	(*N* = 23)	(*N* = 88)		
*Age (years), mean ± SD*	44.1 ± 11.3	39.1 ± 11.6	48.7 ± 8.3	44.8 ± 11.3	0.0011	0.0035
<35	31 (21.1)	14 (38.9)	1 (4.3)	16 (18.2)	0.0118	0.0332
35~44	45 (30.6)	10 (27.8)	6 (26.1)	29 (32.9)		
45~54	45 (30.6)	9 (25.0)	10 (43.5)	26 (29.6)		
≥55	26 (17.7)	3 (8.3)	6 (26.1)	17 (19.3)		
*Body-mass index (kg/m* ^*2*^ *), Mean ± SD*	22.4 ± 2.78	22.6 ± 2.6	22.9 ± 3.1	22.2 ± 2.8	0.7113	0.4724
<18.5	9 (6.1)	2 (5.6)	2 (8.7)	5 (5.7)	0.1709	0.1041
18.5~22.9	80 (54.4)	17 (47.2)	9 (39.1)	54 (61.3)		
23.0~24.9	27 (18.4)	11 (30.6)	3 (13.1)	13 (14.8)		
≥25.0	31 (21.1)	6 (16.7)	9 (39.1)	16 (18.2)		
*Marital status*						
Single	17 (11.6)	8 (22.2)	2 (9.1)	7 (8.0)	0.1989	0.0736
Married	129 (88.4)	28 (77.8)	20 (90.9)	81 (92.0)		
*Menopausal status*						
Premenopausal	96 (65.3)	26 (72.2)	15 (65.2)	55 (62.5)	0.5687	0.5869
Postmenopausal	51 (34.7)	10 (27.8)	8 (34.8)	33 (37.5)		
*Number of children*						
1 or less	32 (24.1)	9 (32.1)	4 (17.4)	19 (23.2)	0.3688	0.6982
2	73 (54.9)	13 (46.4)	15 (65.2)	45 (54.9)		
3 or more	28 (21.1)	6 (21.5)	4 (17.4)	18 (21.9)		
*Education level*						
Middle school or lower	30 (20.6)	5 (13.9)	6 (26.1)	19 (21.8)	0.0151	0.0324
High school	72 (49.3)	13 (36.1)	14 (60.9)	45 (51.7)		
University or higher	44 (30.1)	18 (50.0)	3 (13.0)	23 (26.5)		
*Family income (10,000 won/month)* ^1^						
<199	39 (28.9)	7 (22.6)	7 (33.3)	25 (30.1)	0.5264	0.6247
200~499	62 (45.9)	15 (48.4)	7 (33.3)	40 (48.2)		
≥500	34 (25.2)	9 (29.0)	7 (33.3)	18 (21.7)		
*Oral contraceptive use*						
Never	123 (83.7)	30 (83.3)	19 (82.6)	74 (84.1)	0.9423	0.9835
Ex/current	24 (16.3)	6 (16.7)	4 (17.4)	14 (15.9)		
*Smoking status*						
Never	132 (90.4)	31 (88.6)	22 (95.6)	79 (89.8)	0.3473	0.6355
Ex/current	14 (9.6)	4 (11.4)	1 (4.4)	9 (10.2)		
*Alcohol-drinking status*						
Never	42 (28.8)	11 (31.4)	7 (30.4)	24 (27.3)	0.9362	0.8833
Ex/current	104 (71.2)	24 (95.6)	16 (69.6)	64 (72.7)		
*Oncogenic HPV infection* ^2^						
Negative	41 (27.9)	12 (33.3)	2 (8.7)	27 (30.7)	0.03	0.0787
Positive	106 (72.1)	24 (66.7)	21 (91.3)	61 (69.3)		

Only available variables were used in this study, as not all 147 women completed the entire questionnaire. This table presents the number of subjects and their percentages (mean ± SD). ^1^The won-dollar exchange rate was approximately 1,280 won (per dollar) in 2002. ^2^Oncogenic HPV infection status was determined by measurement of 13 oncogenic HPV DNA types using Hybrid Capture 2. ^3^Abortion was subclassified as spontaneous abortion and induced abortion. ^4^Chi-square and *t*-tests were used to assess the differences in the categorical and continuous variables, respectively, between nonabortion and spontaneous abortion; ^5^Chi-square test and ANOVA were used to assess the differences in the categorical and continuous variables, respectively, among nonabortion, spontaneous abortion, and induced abortion; *P* < 0.05.

**Table 2:** Distribution of the species averaged across the nonabortion, spontaneous abortion, and induced abortion groups.

Bacteria	Nonabortion (%)	Spontaneous abortion (%)	Induced abortion (%)
*Atopobium vaginae*	4.232	23.282^*∗*^	6.867
*Megasphaera *spp.	1.029	4.608^*∗*^	1.556
*Gardnerella vaginalis*	0.130	3.579^*∗*^	1.811
*Leptotrichia amnionii*	0.063	1.928^*∗*^	1.436^*∗*^
*Sneathia sanguinegens*	0.003	0.549^*∗*^	1.932

Only the species which were significantly increased were presented; ^*∗*^*P* < 0.05 Wilcoxon rank sum test compared with women with nonabortion.

**Table 3:** Factor loadings determined by principal component analysis.

Bacteria	Factor 1	Factor 2	Factor 3	Factor 4	Factor 5	Factor 6	Factor 7
*AY959069_s*	0.404	−0.007	−0.006	−0.008	0.002	−0.055	−0.062
*Lactobacillus fornicalis*	−0.133	−0.04	−0.032	0.639	−0.09	−0.07	−0.06
*AY958888_s*	0.613	−0.013	−0.031	0.011	−0.02	−0.052	−0.005
*Leptotrichia amnionii*	0.4	−0.007	0.293	−0.051	−0.06	0.124	−0.082
*AY959109s*	0.741	−0.009	−0.033	0.003	0.011	0.026	0.021
*Prevotella timonensis*	0.414	0.0077	0.01	−0.028	0.019	0.062	−0.068
*Prevotella amnii*	0.528	0.0242	0.05	0.047	−0.01	0.103	−0.032
*AY958940_s*	0.631	0.0043	0.032	0.029	−0.03	0.046	−0.018
*Megasphaera*	0.711	−0.022	−0.042	−0.026	−0.05	−0.066	−0.006
*AY959023*	0.612	−0.011	−0.026	0.016	−0.03	−0.114	−0.009
*P003395_s*	0.525	−0.009	−0.053	0.038	−0.02	−0.143	0.023
*DQ666092_s*	0.215	−0.02	0.006	−0.041	−0.07	−0.039	0.067
*Sneathia_sanguinegens*	0.208	−0.002	−8*E* − 04	−0.002	−0.01	0.027	−0.032
*AY995258_s*	0.158	−0.003	0.016	0.016	−0.02	−0.03	0.026
*Escherichia coli*	−0.039	0.9649	−0.012	−0.004	−0.08	−0.056	−0.01
*Streptococcus anginosus*	−0.018	0.5555	0.013	0.011	0.328	0.083	−0.02
*Escherichia fergusonii*	−0.036	0.9649	−0.011	−5*E* − 04	−0.08	−0.055	−0.011
*Peptostreptococcus anaerobius*	−0.032	−0.006	0.986	0.011	−0.01	−0.045	−0.008
*Peptoniphilus indolicus*	−0.025	−0.003	0.987	0.009	−0.01	−0.044	−0.012
*Pseudomonas trivialis*	−0.094	−0.027	−0.027	−0.156	−0.07	0.016	−0.01
*Lactobacillus psittaci*	−0.13	−0.034	−0.034	0.773	−0.11	−0.05	−0.003
*Lactobacillus jensenii*	−0.115	−0.008	−0.028	0.7	−0.06	−0.025	0.004
*Mycoplasma hominis*	−0.025	0.1032	0.003	0.029	0.58	0.086	0.066
*Lactobacillus gasseri*	−0.04	−0.061	−0.009	−0.027	0.649	−0.071	−0.062
*Lactobacillus acidophilus*	−0.025	0.025	−0.001	0.019	0.774	0.049	−0.086
*Aerococcus christensenii*	−0.072	−0.008	−0.022	−0.058	0.04	0.33	0.123
*Dialister micraerophilus*	0.146	0.0147	0.014	0.048	−0	0.822	−0.025
*Staphylococcus epidermidis*	−0.06	−0.01	−0.008	−0.012	−0.01	0.277	−0.027
*Enterococcus faecalis*	−0.066	−0.003	−0.005	0.037	−0.05	0.704	−0.068
*Lactobacillus iners*	−0.192	−0.105	−0.073	−0.452	−0.26	−0.102	−0.209
*Lactobacillus crispatus*	−0.298	−0.062	−0.076	0.183	−0.06	−0.28	−0.415
*Atopobium vaginae*	0.259	−0.03	−0.021	0.192	−0.07	0.146	0.334
*Prevotella bivia*	−0.031	−0.015	−0.024	0.041	0.182	0.022	0.312
*Lactobacillus vaginalis*	−0.191	−0.058	−0.056	0.325	−0.04	−0.225	−0.375
*Gardnerella vaginalis*	−0.076	−0.02	−0.011	−0.012	0.032	−0.132	0.719
*Ureaplasma parvum*	−0.077	−0.013	−0.016	−0.01	0.519	−0.069	0.147
*Streptococcus agalactiae*	−0.084	−0.012	−0.015	−0.062	−0.03	0.099	0.143
*Prevotella denticola*	−0.066	−0.008	−0.001	−0.009	−0.07	−0.126	0.61
*Ureaplasma urealyticum*	−0.046	−0.008	−0.004	−0.021	−0.03	0.005	0.037
*Pseudomonas cedrina*	−0.043	−0.011	−0.001	−0.06	−0.02	−0.018	0.034
*Microbacterium hydrocarbonoxydan*	−0.05	−0.016	−0.011	0.093	−0.05	−0.022	−0.007
*Streptococcus pseudopneumoniae*	−0.074	0.0159	−0.012	−0.078	0.051	0.011	0.093
*Lactobacillus johnsonii*	−0.048	−0.008	−0.007	−0.059	0.078	−0.028	−0.002
*Microbacterium ginsengisoli*	−0.037	−0.006	−0.005	−0.045	−0.02	−0.007	0.01
*Streptococcus salivarius*	−0.049	0.0005	−0.008	−0.056	−0.02	−0.012	0.022

Principal component analysis was performed for all 45 microbial species. A total of seven factors showing an eigenvalue > 1.5 were identified, and the factor-loading values of 45 species in each factor were calculated. − means negative correlation.

**Table 4:** Rank correlations between epidemiological factors and relative abundances of microbial species.

Epidemiological factors	Bacteria	Coefficient	*P* value
Age group	*Aerococcus christensenii*	0.1343592	0.027
Age group	*Peptoniphilus indolicus*	0.1209233	0.008
Age group	*Pseudomonas trivialis*	0.0916399	0.038
Age group	*Streptococcus agalactiae*	0.0877354	0.021
Age group	*Peptostreptococcus anaerobius*	0.0625861	0.046
Age group	*Lactobacillus psittaci*	−0.107028	0.045
Age group	*Lactobacillus vaginalis*	−0.1264355	0.011
Alcohol-drinking status	*Peptostreptococcus anaerobius*	−0.1295681	0.033
Alcohol-drinking status	*Peptoniphilus indolicus*	−0.1548173	0.042
Duration of alcohol drinking	*Ureaplasma parvum*	0.1506007	0.034
Duration of alcohol drinking	*Peptostreptococcus anaerobius*	−0.0775172	0.037
Frequency of alcohol drinking	*Peptostreptococcus anaerobius*	−0.0852376	0.024
Frequency of alcohol drinking	*Aerococcus christensenii*	−0.141315	0.023
BMI group	*Streptococcus agalactiae*	0.0861567	0.041
Breast feeding status	*Microbacterium hydrocarbonoxydan*	0.0943396	0.001
Breast feeding status	*Escherichia fergusonii*	0.0566038	0.013
Breast feeding status	*Lactobacillus acidophilus*	0.0471698	0.023
Number of breast feedings	*Lactobacillus acidophilus*	0.064167	0.034
Number of children	*Streptococcus anginosus*	0.115846	0.029
Education group	*Lactobacillus fornicalis*	0.1377228	0.032
Education group	*Lactobacillus jensenii*	0.1368603	0.007
Education group	*Lactobacillus vaginalis*	0.1236343	0.031
Education group	*Lactobacillus acidophilus*	−0.0715929	0.028
First parity age	*Lactobacillus acidophilus*	−0.0587645	0.031
Heavy physical days	*AY995258_s*	−0.0465116	0.013
Heavy physical days	*Pseudomonas cedrina*	−0.0775194	0.001
Heavy physical days	*Lactobacillus crispatus*	−0.250918	0.025
Family income level group	*Prevotella bivia*	0.1919973	0.002
Family income level group	*Lactobacillus crispatus*	0.169805	0.027
Family income level group	*Lactobacillus fornicalis*	0.1585407	0.014
Family income level group	*Lactobacillus jensenii*	0.1482851	0.004
Family income level group	*Lactobacillus psittaci*	0.1410558	0.019
Family income level group	*Sneathia sanguinegens*	−0.1516476	0.006
Induced abortion	*Leptotrichia amnionii*	0.1887626	0.006
Induced abortion	*AY958940 s*	0.1171086	0.016
Number of induced abortions	*Leptotrichia amnionii*	0.1085734	0.041
Medium physical days	*Pseudomonas trivialis*	0.1102151	0.039
Menarche age	*AY995258_s*	−0.0666989	0.012
Menstrual cycle	*Pseudomonas trivialis*	0.1332942	0.013
Menstrual cycle	*Lactobacillus fornicalis*	−0.1884909	0.01
Menstrual regulation	*AY959069_s*	−0.1232877	0.002
Menstrual regulation	*AY959023_s*	−0.1438356	0.03
Menstrual regulation	*Leptotrichia amnionii*	−0.1637609	0.024
Mensday regulation	*Streptococcus agalactiae*	−0.0625	0.023
Mensday regulation	*Pseudomonas trivialis*	−0.1125	0.002
Mensday regulation	*AY959069_s*	−0.1125	0.002
Mensday regulation	*Streptococcus pseudopneumoniae*	−0.1125	0.002
Mensday regulation	*Ureaplasma urealyticum*	−0.1375	0
Mensday regulation	*Prevotella amnii*	−0.15	0
Mensday regulation	*_P003395_s*	−0.1625	0
Mensday regulation	*Lactobacillus vaginalis*	−0.2163462	0.03
Mensday regulation	*Aerococcus christensenii*	−0.2269231	0.021
Mensday	*_P003395_s*	−0.1732194	0.009
Mensday	*Prevotella timonensis*	−0.2017094	0.019
Oral contraceptive use	*Staphylococcus epidermidis*	−0.1	0.04
Oral contraceptive use	*Streptococcus anginosus*	−0.1532258	0
Sitting time	*Escherichia fergusonii*	0.0714913	0.023
Sitting time	*Microbacterium hydrocarbonoxydan*	−0.0790167	0.04
Sitting time	*Pseudomonas trivialis*	−0.1163928	0.009
Smoking	*Escherichia fergusonii*	−0.0451128	0.013
Smoking	*Ureaplasma urealyticum*	−0.1654135	0
Smoking	*Aerococcus christensenii*	−0.2230576	0.035
Spontaneous abortion	*Atopobium vaginae*	0.3357488	0.026
Spontaneous abortion	*Sneathia sanguinegens*	0.3115942	0.009
Spontaneous abortion	*AY959109_s*	0.3007246	0.015
Spontaneous abortion	*Gardnerella vaginalis*	0.2862319	0.045
Spontaneous abortion	*Leptotrichia amnionii*	0.2657005	0.024
Spontaneous abortion	*AY958940_s*	0.2258454	0.021
Spontaneous abortion	*Streptococcus pseudopneumoniae*	−0.1594203	0.043
Spontaneous abortion	*Lactobacillus fornicalis*	−0.2222222	0.049
Spontaneous abortion	*Ureaplasma urealyticum*	−0.25	0.001
Number of spontaneous abortions	*Atopobium vaginae*	0.2729167	0.045
Number of spontaneous abortions	*AY959109_s*	0.2552083	0.017
Number of spontaneous abortions	*Sneathia sanguinegens*	0.2416667	0.026
Number of spontaneous abortions	*Leptotrichia amnionii*	0.2125	0.045
Number of spontaneous abortions	*AY958940_s*	0.178125	0.042
Number of spontaneous abortions	*Staphylococcus epidermidis*	−0.1239583	0.042
Number of spontaneous abortions	*Lactobacillus fornicalis*	−0.2	0.045
Number of spontaneous abortions	*Ureaplasma urealyticum*	−0.215625	0.001
Walking hours per day	*Lactobacillus iners*	0.1760537	0.016
Walking hours per day	*Pseudomonas trivialis*	−0.1173691	0.009

Forty-five (45) species filtered for the 0.1%-or-over rate among total species identified using pyrosequencing. Continuous variables (age, BMI, first parity age, walking day for 10 min or longer per week, menstrual day, menstrual cycle, and duration of oral contraceptive use) and categorical variables (marital status, education level, family income level, smoking frequency, duration of smoking, alcohol drinking, alcohol-drinking frequency, spontaneous abortion, number of spontaneous abortions, and number of children) of 27 factors were used for the analysis. Somers' *D* rank correlation coefficients were measured and presented. *P* < 0.05 was considered to be significant. − means negative correlation.

**Table 5:** High-risk microbial patterns associated with spontaneous abortion and induced abortion.

Microbiota	Groups	Nonabortion (%)	Spontaneous abortion (%)	Induced abortion (%)	Univariate (ORs, 95% CI)		Multivariate (ORs, 95% CI)	
					Spontaneous abortion	Induced abortion	Spontaneous abortion	Induced abortion
Factor 1	Low	29 (80.6)	11 (47.8)	60 (68.2)	1 (ref.)	1 (ref.)	1 (ref.)	1 (ref.)
	High	7 (19.4)	12 (52.2)	28 (31.8)	4.51 (1.41–14.5)	1.93 (0.76–4.95)	16.4 (1.88–42.5)	2.63 (0.87–7.92)
*Atopobium vaginae*	Low	29 (80.6)	10 (43.5)	61 (69.3)	1 (ref.)	1 (ref.)	1 (ref.)	1 (ref.)
	High	7 (19.4)	13 (56.5)	27 (30.7)	5.38 (1.68–17.29)	1.83 (0.72–4.7)	11.27 (1.57–81.00)	2.42 (0.82–7.16)
*Leptotrichia amnionii*	Low	32 (88.9)	15 (65.2)	63 (74.9)	1 (ref.)	1 (ref.)	1 (ref.)	1 (ref.)
	High	4 (11.1)	8 (34.8)	25 (28.1)	4.26 (1.11–16.42)	3.17 (1.02–9.91)	11.47 (1.22–107.94)	4.17 (1.05–16.49)
*Sneathia sanguinegens*	Low	32 (88.9)	14 (60.9)	74 (84.1)	1 (ref.)	1 (ref.)	1 (ref.)	1 (ref.)
	High	4 (11.1)	9 (39.1)	14 (15.9)	5.14 (1.35–19.54)	1.51 (0.46–4.95)	6.89 (1.07–44.33)	1.41 (0.39–5.05)
*Megasphaera *spp.	Low	31 (86.1)	15 (65.2)	71 (80.7)	1 (ref.)	1 (ref.)	1 (ref.)	1 (ref.)
	High	5 (13.9)	8 (34.8)	17 (19.3)	3.30 (0.92–11.85)	1.48 (0.5–4.38)	4.99 (0.81–30.82)	2.61 (0.71–9.57)
*Gardnerella vaginalis*	Low	29 (80.6)	14 (60.8)	59 (67.1)	1 (ref.)	1 (ref.)	1 (ref.)	1 (ref.)
	High	7 (19.4)	9 (39.2)	29 (32.9)	2.66 (0.82–8.63)	2.03 (0.8–5.2)	1.16 (0.16–8.32)	2.47 (0.83–7.32)

ORs, 95% CIs were calculated by multivariate logistic regression after controlling for age, menopause, BMI, smoking, alcohol, and HPV. The low- and high-abundance microbiota were defined by the low tertile and high tertile, respectively.
