# Supplementary material for: High Prevalence of Leptotrichia amnionii, Atopobium vaginae, Sneathia sanguinegens, and Factor 1 Microbes and Association of Spontaneous Abortion among Korean Women
Source: Biomed Res Int. 2017 Dec 13;2017:5435089. doi: 10.1155/2017/5435089 (PMC5745682; doi:10.1155/2017/5435089)
Supplement: Supplementary file 1 — Supplementary table 1: Distribution of vaginal bacteria from non-abortion, spontaneous abortion and induced abortion women in Korea. Supplementary figure 1: Shannon index (a) and Chao 1 (b) index across non-abortion, spontaneous abortion and induced abortion women. [file 5435089.f1.pdf]

Supplementary table 1: **Distribution of the species averaged across the non-abortion, spontaneous abortion and induced abortion groups**

Bacteria	Non abortion (%)	Spontaneous Abortion (%)	Induced abortion (%)
Lactobacillus iners	26.210	20.323	28.012
Lactobacillus crispatus	25.291	15.858	20.482
Pseudomonas trivialis	4.303	3.221	3.711
Atopobium vaginae	4.232	23.282*	6.867
Streptococcus agalactiae	4.073	1.873	0.161
Prevotella bivia	3.372	2.999	4.465
Microbacterium ginsengisoli	2.718	0.003	0.000
Staphylococcus epidermidis	2.544	0.000	0.077
Aerococcus christensenii	2.375	1.759	2.322
Pseudomonas cedrina	2.256	0.025	0.057
Ureaplasma parvum	2.062	0.405	2.193
Streptococcus pseudopneumoniae	1.870	0.000	0.114
Streptococcus salivarius	1.523	0.000	0.098
Lactobacillus jensenii	1.481	0.440	0.502
Lactobacillus gasseri	1.476	0.002	0.821
Microbacterium hydrocarbonoxydan	1.194	0.013	0.012
Lactobacillus fornicalis	1.067	0.553	4.251
Streptococcus anginosus	1.044	0.001	0.286
Megasphaera spp.	1.029	4.608*	1.556
Mycoplasma hominis	0.533	0.617	1.215
Prevotella timonensis	0.352	1.581	0.911
AY958888	0.278	1.464*	1.103
Lactobacillus psittaci	0.183	0.031	0.188
Dialister micraerophilus	0.181	0.206	0.091
Gardnerella vaginalis	0.130	3.579*	1.811
AY959109	0.117	0.337*	0.298
Lactobacillus vaginalis	0.094	0.202	0.100
Ureaplasma urealyticum	0.078	0.000*	0.584
AY959023	0.066	2.102	0.993
Leptotrichia amnionii	0.063	1.928*	1.436*
Peptoniphilus indolicus	0.037	1.350	0.053
Escherichia coli	0.035	0.003	0.642
AY958940	0.027	0.120*	0.092
Prevotella amnii	0.026	1.049	1.729
DQ666092	0.016	0.472	0.033
Sneathia sanguinegens	0.003	0.549*	1.932
P003395	0.002	3.005	0.616
Escherichia fergusonii	0.002	0	0.367

AY959069	0.002	0.662	1.483
AY995258	0.001	1.187	0.023
Enterococcus faecalis	0.001	0.001	1.155
Peptostreptococcus anaerobius	0.000	1.979	0.040
Lactobacillus acidophilus	0.000	0.000	0.251
Lactobacillus johnsonii	0.000	0	1.926
Prevotella denticola	0.000	0	0.463

Only >0.1% of the major species among the study subjects are presented.
* $P < 0.05$ Wilcoxon rank sum test compared with women with non-abortion

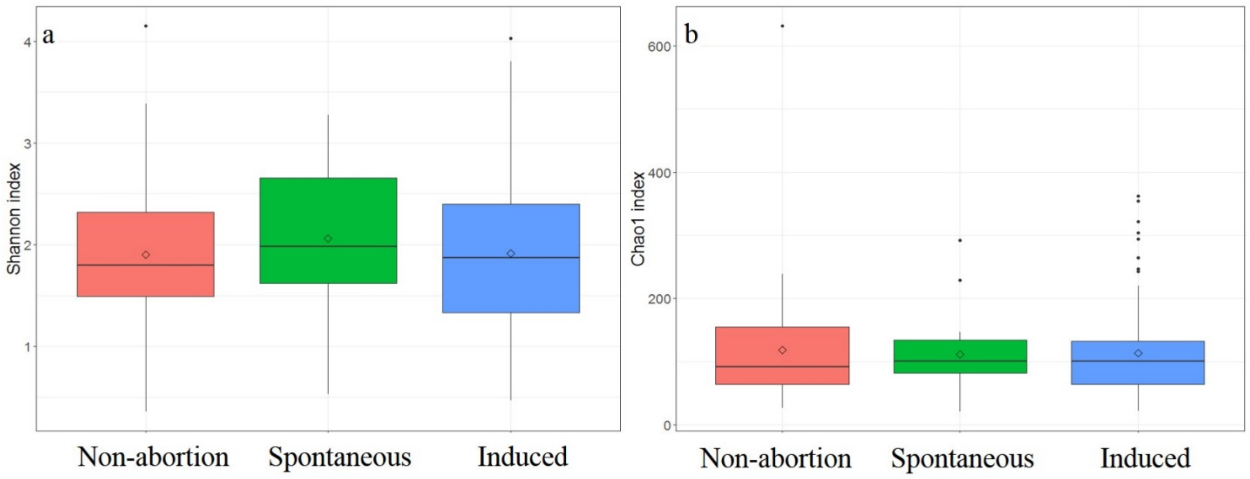

Supplementary figure 1. Shannon index (a) and Chao 1 (b) index were used to measure alpha diversity.
